# Integrating Physical Health Dimensions in Middle-Aged and Older Adults to Predict Drug Misuse and Relapse Through a Comprehensive Needs Model: Protocol for a Mixed Methods Study

**DOI:** 10.2196/88987

**Published:** 2026-03-19

**Authors:** Ema Madyaningrum, Eny Purwandary, Supriyati Supriyati, Ariani Arista Putri Pertiwi, Made Satya Nugraha Gautama, Nurlaela Widyarini

**Affiliations:** 1 Department of Mental Health and Community Nursing Faculty of Medicine, Public Health, and Nursing Universitas Gadjah Mada Sleman, Yogyakarta Indonesia; 2 Faculty of Psychology, Universitas Muhammadiyah Surakarta, Surakarta, Indonesia Surakarta, Center of Java Indonesia; 3 Department of Health Behavior, Environment, and Social Medicine, Faculty of Medicine, Public Health and Nursing, Universitas Gadjah, Yogyakarta, Indonesia Sleman, Yogyakarta Indonesia; 4 Department of Basic Nursing and Emergency; Faculty of Medicine, Public Health, and Nursing; Universitas Gadjah Mada, Yogyakarta, Indonesia Sleman, Yogayakrta Indonesia; 5 Department of Nursing, Faculty of Medicine, Universitas Pendidikan Ganesha, Singaraja, Indonesia Singaraja, Bali Indonesia; 6 Faculty of Psychology, Universitas Muhammadiyah Jember, Jember, Indonesia Jember, East Java Indonesia

**Keywords:** substance abuse, relapse, middle age, older adults, health belief model

## Abstract

**Background:**

Drug misuse poses significant health risks, with rising prevalence globally, particularly affecting middle-aged and older adults. In Indonesia, drug misuse has complex impacts across physical, psychological, social, and spiritual dimensions. Addressing these issues is crucial as Indonesia transitions to an aging population.

**Objective:**

This study protocol aims to develop a comprehensive model incorporating physical health dimensions to predict drug misuse and relapse among middle-aged and older adults, grounded in the health belief model.

**Methods:**

We will use a sequential mixed methods design, combining quantitative data from national databases and medical records with qualitative insights from stakeholders. The respondents will consist of three groups: (1) individuals with substance use disorders recruited from the Badan Narkotika Nasional (National Narcotics Agency), (2) participants from *lembaga pemasyarakatan* (penitentiary institutions) and *balai pemasyarakatan* (community correction centers), and (3) stakeholders involved in drug rehabilitation services and experts in the field of education. Data analysis will be conducted in sequential stages, including descriptive statistics, bivariate analysis, and logistic regression to identify predictors of relapse. Qualitative data will be analyzed using thematic analysis to explore stakeholders’ perspectives.

**Results:**

This study was funded by the Ministry of Education, Culture, Research, and Technology of Indonesia in 2024. Data collection started in July 2024 and ended in December 2025. The study is structured into 3 phases. Upon completion, it is expected to support the development of a comprehensive model incorporating physical health dimensions to predict drug misuse and relapse among middle-aged and older adults, grounded in the health belief model. The findings are expected to be published by the end of 2026.

**Conclusions:**

The study is expected to identify key health status indicators and levels of readiness to change for the prevention of drug misuse relapse, as well as to develop a preventive model that addresses the specific needs of vulnerable populations, thereby enhancing the effectiveness of intervention strategies. By using a holistic approach to address the multifaceted impacts of drug misuse, this study will aim to improve health outcomes for middle-aged and older adults.

**International Registered Report Identifier (IRRID):**

DERR1-10.2196/88987

## Introduction

People who misuse drugs are among the most vulnerable populations for health issues, with their numbers continuing to rise. Globally, from 2011 to 2021, there was an estimated 5.85% increase in drug misuse, reaching approximately 296 million people [1]. In Indonesia, the prevalence of people who misuse drugs was 1.95% as of 2021 [1]. The types of drugs used are influenced by age, socioeconomic status, and prevailing drug trends at a given time. Illicit drugs commonly used by individuals in Indonesia include cannabis, amphetamines, and nitrazepam (known locally as *nipam* or *koplo*) [2].

Drug misuse leads to complex physical, psychological, social, and spiritual impacts. Physically, it can cause overdoses, drug poisoning, sexually transmitted diseases, liver cirrhosis, cancer, hypertension, and respiratory diseases. In Indonesia, 65.8% of users experience severe addiction [3]. Psychologically, it may result in anxiety, depression, suicidal thoughts, sleep disorders, and posttraumatic stress disorder [4-7]. Social impacts include strained family relationships, divorce, social stigma, and legal issues [8,9]. Spiritually, there is often a decline in religious engagement [4].

Drug misuse is prevalent among individuals aged 15 to 64 years, with adolescents traditionally representing the highest-use demographic [1]. However, there is a growing number of users in older age groups [10-12]. In Indonesia, adults aged 25 to 49 years are the most affected [2], whereas in the United States, opioid use is high among women aged 68 to 77 years [13]. In a previous study in Indonesia, 11.27% of middle-aged and older adults from rehabilitation centers reported drug misuse [3]. Such trends highlight the need to focus on different at-risk groups, especially as Indonesia transitions to an older population structure, with more than 10% of its population comprising older adults since 2021. This shift necessitates addressing the health challenges of an aging population, including the rising number of older people who misuse drugs, which can degrade their overall health status. To improve life quality for older adults, it is crucial to prevent drug misuse. A holistic prevention strategy addressing physical, psychological, social, and spiritual needs is essential [14-16]. Since 2016, Indonesia’s National Narcotics Agency (Badan Narkotika Nasional; BNN) has developed the Drugs Rehabilitation Information System (Sistem Rehabilitasi Narkoba; SIRENA) to provide real-time updates on drug misuse nationally, aiding in intervention development.

Using the extensive data available from this system offers a significant opportunity to advance our understanding of drug misuse relapse prevention and to better identify the health care needs of vulnerable older adult populations. As individuals age, they encounter a multitude of health challenges, ranging from chronic illnesses to mobility issues, which can be further complicated by drug misuse. This interrelation underscores the critical need for focused and detailed research aimed specifically at these groups. This study aims to explore several key areas regarding drug misuse among middle-aged and older adult populations. First, it seeks to identify the physical health profile of people who misuse drugs within these groups. Second, the study will examine the risk and protective factors related to physical and biological health that may influence drug misuse relapse. Furthermore, it aims to determine the specific physical and biological health needs that can prevent relapse and anticipate drug misuse among middle-aged and older adults. Finally, the study will intend to develop a health model based on the health belief model to effectively prevent relapse in these vulnerable populations.

## Methods

### Study Design

**Figure 1:**
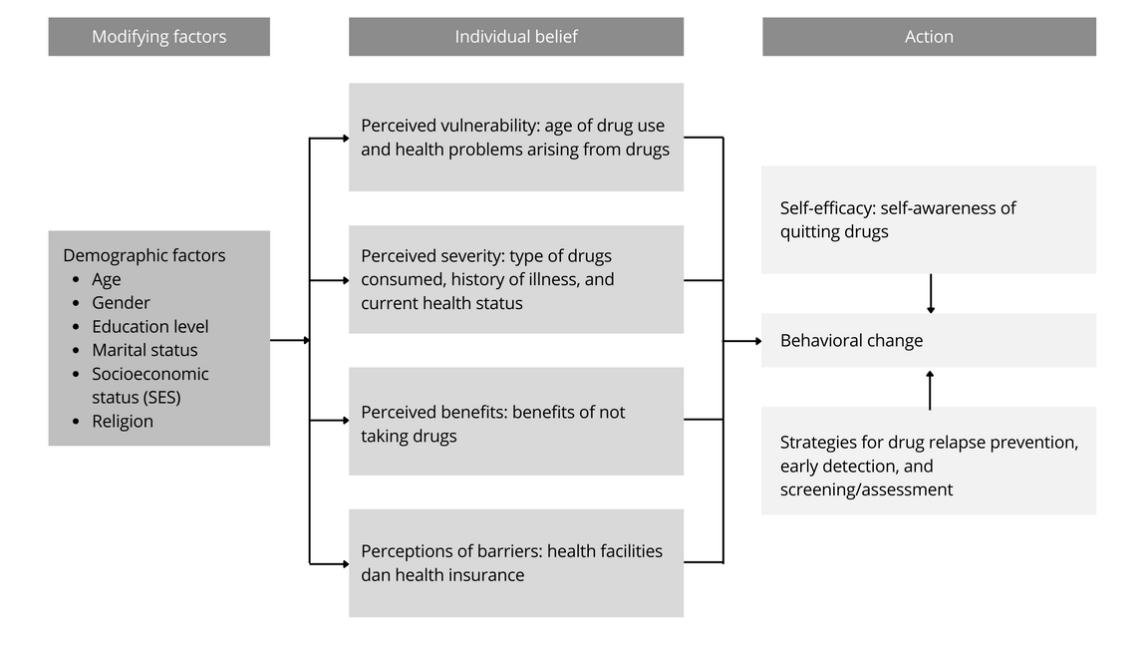
Approach based on the health belief model.

This research will use a sequential mixed methods design, combining quantitative and qualitative research approaches [17] using the health belief model approach (Figure 1). The results from the quantitative phase will inform and guide the implementation of the qualitative phase in the next stage of the study [16].

### Participants and Recruitment

#### Phase 1 and 2 (Quantitative Phase)

A total of 355 records will be assessed from the BNN. Furthermore, 381 records will be assessed from penitentiary institutions or *lembaga pemasyarakatan* (LPs) and community correction centers or *balai pemasyarakatan* (BPs). For the quantitative analysis, we used secondary data from the Indonesian government’s drug rehabilitation records. These resources originate from the BNN, which serves as the central agency for narcotics regulation; LPs, which provide drug rehabilitation services to prisoners who have been criminalized for drug-related offenses; and BPs, which offer rehabilitation for individuals preparing to reintegrate into their communities.

The database for the years 2022 to 2024 contains detailed information on individuals involved in drug misuse, the services they received, and their health status as recorded in medical records. The medical records will focus on physical health assessments, treatment interventions, and instances of relapse.

#### Inclusion and Exclusion Criteria

Participant eligibility criteria are presented in Textbox 1.

**Participant eligibility criteria.**

**Inclusion criteria**

- People with a history of drug misuse aged ≥40 years who have previously visited polyclinics or been hospitalized for drug-related problems
- People with a record of health checks
- People with documentation of any illnesses that occurred during their rehabilitation or recovery process

**Exclusion criteria**

- Incomplete medical record data

The sampling technique used in this study is total sampling, whereby all data that meet the inclusion and exclusion criteria will be included in the analysis.

#### Phase 3 (Qualitative Phase)

For the qualitative data collection, participants will be selected using purposive sampling techniques. This selection will focus on individuals who have expertise or experience in dealing with drug misuse–related issues. Participants will include stakeholders such as health workers (doctors and nurses) and rehabilitation officers. Their involvement will be determined based on recommendations from the rehabilitation leader of the Provincial National Narcotics Board, ensuring that the participants have relevant knowledge and insights related to the study’s focus. The interviews will take place either in person or via videoconferencing and will last approximately 60 to 90 minutes. With participants’ consent, each interview will be audio-recorded and then transcribed for detailed analysis.

### Variables and Measurements

#### Phase 1

The first phase aims to explore several key aspects of drug misuse among middle-aged and older adult populations. Specifically, it seeks to identify the physical health profiles of individuals with substance use disorders within these age groups. On the basis of this objective, the study further examines participants’ perceptions of their own health status.

To understand the influence of various factors on self-health perception, we apply the health belief model. The modifying factors include demographic variables such as age, gender, education, occupation, ethnicity, marital status, living arrangements, and religion. The perceived susceptibility factors include being a drug dealer, types of drugs used, routine drug misuse, excessive drug misuse, and the impact of these behaviors on occupational functioning and interpersonal relationships. Examining these variables enables the identification of behavioral patterns and their impact on self-assessment of health. The perceived severity variables include length of conviction, recidivism status, and excessive drug misuse.

#### Phase 2

The second phase aims to examine risk and protective factors related to physical and biological health that may influence drug misuse relapse. Prediction of relapse will be assessed through readiness to change for discontinuing drug misuse. The readiness to change is determined by the application of the transtheoretical model, which explains the principle of change, specifically behavioral change [18,19]. This variable is measured by the University of Rhode Island Change Assessment Scale [20]. The University of Rhode Island Change Assessment Scale has been used to assess people who participated in the rehabilitation program at the BNN [21]. From the total score, the variable will be categorized to precontemplation (score <8), contemplation (8-11), action (11-14), and maintenance (>14).

The independent variables include the demographics of respondents, such as gender, age, education background, marital status, employment status, and religion. Other variables include drug choice; the Addiction Severity Index, Fifth Edition [22]; risk for substance use as measured by the Alcohol, Smoking, and Substance Involvement Screening Test [23]; and rehabilitation monitoring, which encompasses health status, psychological status, social status, and environmental status.

#### Phase 3

Finally, the study aims to develop a health model grounded in the health belief model to effectively prevent relapse among these vulnerable populations.

### Statistical Analysis

#### Phases 1 and 2

Descriptive statistics will be used to summarize demographic characteristics, substance use history, patterns of drug misuse, and health perceptions among individuals with substance use disorders, and bivariate analyses will be conducted to examine associations between independent and dependent variables. Furthermore, inferential statistical analyses, specifically logistic regression, will be performed to identify predictors of health outcomes and drug misuse relapse while controlling for demographic variables. A *P* value <.05 will be considered the threshold for statistical significance throughout the analysis.

#### Phase 3

Thematic analysis will be conducted to identify and analyze patterns within the qualitative data gathered from interviews. Transcripts will be coded inductively to develop themes that reflect participants’ experiences and insights. NVivo software (Lumivero) will be used to assist in the organization and analysis of qualitative data.

### Ethical Considerations

Ethics approval was obtained from the institutional review board of Medical Health Research Ethics Committee of Faculty of Medicine, Public Health, and Nursing Universitas Gadjah Mada—Dr Sardjito General Hospital (ref KE/FK/1652/EC/2024). Informed consent will be secured from all interview participants, emphasizing the voluntary nature of participation and the right to withdraw at any time. All data will be treated confidentially to ensure participant anonymity.

## Results

This study was funded by the Ministry of Education, Culture, Research and Technology of Indonesia in 2024. Data collection started in July 2024 and ended in December 2025. The study is structured into 3 phases. In the last phase of the study, findings from phases 1 and 2 will be used to develop a comprehensive model incorporating physical health dimensions to predict drug misuse and relapse among middle-aged and older adults, grounded in the health belief model. Currently, the research is in the data analysis phase, and the publication of findings is anticipated by the end of 2026.

## Discussion

### Anticipated Findings

This study aims to develop a comprehensive model incorporating physical health dimensions to predict drug misuse and relapse among middle-aged and older adults, grounded in the health belief model. In the initial phase, the study hypothesizes that illicit drug misuse is associated with perceptions of health status. This phase will also identify factors that influence the health status of individuals who use illicit drugs. In the second phase, the study will assess the readiness of people who misuse drugs to undergo rehabilitation and discontinue illicit drug use. In the final phase, the findings from the previous stages will be integrated to develop a comprehensive model incorporating physical health dimensions to predict drug misuse and relapse among middle-aged and older adults.

This study protocol outlines the approach to developing a comprehensive strategy for drug misuse prevention and treatment among middle-aged and older adults. Recognizing the rising prevalence of drug misuse among these age groups, particularly in Indonesia, this study will target specific interventions in response to demographic shifts.

The Indonesian government has 4 formal drug rehabilitation institutions: government hospitals, LPs, BPs, and the BNN. Although they collaborate in providing drug rehabilitation to people with substance use disorders, they each have different responsibilities. LPs are responsible for drug rehabilitation and social rehabilitation of prisoners who have legal problems related to narcotic use, such as narcotic distributors, sellers, and dealers, after which they will be returned to society. BPs accompany them during the preparation process for returning to society. The next institution is BNN, whose drug misuse rehabilitation service includes inpatient and outpatient services for people who misuse drugs with mild health problems. The last institution is hospitals. people who misuse drugs with health-related problems or drug addition receive more services from hospitals [24,25].

In our study, we will use a mixed methods approach to thoroughly investigate the factors influencing drug misuse, including demographic, behavioral, and psychosocial elements. By incorporating physical health dimensions into our model, we aim to provide a detailed understanding of self-health perception and its impact on drug misuse patterns. The logistic regression analysis will help identify significant predictors of relapse, offering a basis for tailored intervention strategies. Additionally, by involving stakeholders such as health care providers and rehabilitation officers, the study will ground its findings in practical experience, ensuring the proposed health model addresses real-world needs and challenges.

As Indonesia’s population ages, addressing health issues related to drug misuse among middle-aged and older adults will become increasingly vital. This study will inform public health policies and intervention programs, aiming to improve their effectiveness and relevance. By prioritizing comprehensive needs and a holistic approach, this protocol aims to pave the way for improving the quality of life for those at risk of drug misuse and relapse.

### Strengths and Limitations

A major strength of this study is the involvement of multiple institutions engaged in the rehabilitation of individuals with drug misuse disorders. This multi-institutional approach enables a more comprehensive understanding of behavioral changes across different rehabilitation settings.

The limitation of this study is the restricted availability of variables related to the health status of individuals with substance use disorders, particularly data obtained from LPs and BPs. Additionally, a substantial proportion of health data from individuals undergoing rehabilitation at the BNN are incomplete because the implementation of standardized and comprehensive rehabilitation health assessments was still in its early stages in 2024.

### Conclusions

The study is expected to identify key health status indicators and levels of readiness to change for the prevention of drug misuse relapse, as well as to develop a preventive model that addresses the specific needs of vulnerable populations, thereby enhancing the effectiveness of intervention strategies. By using a holistic approach to address the multifaceted impacts of drug misuse, this study will aim to improve health outcomes for middle-aged and older adults with a history of drug misuse.

### Funding

This study was supported by research grants from Ministry of Education, Culture, Research, and Technology of Indonesia.

### Data Availability

The datasets generated or analyzed during this study are available from the corresponding author on reasonable request.

## Abbreviations

BP: balai pemasyarakata
BNN: Badan Narkotika Nasional
LP: lembaga pemasyarakatan
SIRENA: Sistem Rehabilitasi Narkoba (Drugs Rehabilitation Information System)
